# Prevalence and risk factors of cognitive impairment in patients with coronary heart disease: a systematic review and meta-analysis

**DOI:** 10.3389/fcvm.2026.1754515

**Published:** 2026-05-07

**Authors:** Jie Han, Bing Xie, Na Zhao

**Affiliations:** 1 School of Nursing, Changzhi Medical College, Changzhi, Shanxi, China; 2 Department of Nursing, Heping Hospital Affiliated to Changzhi Medical College, Changzhi, Shanxi, China

**Keywords:** cognitive impairment, coronary heart disease, influencing factors, meta-analysis, prevalence

## Abstract

**Background:**

The prevalence and risk factors of cognitive impairment in patients with coronary atherosclerotic heart disease (CHD) vary substantially depending on the measurement approaches and time points adopted. Through a meta-analysis, we aim to clarify the prevalence and risk factors of cognitive impairment in CHD patients, thereby providing a reference for the implementation of targeted interventions.

**Methods:**

Computerized searches were conducted across 9 databases, including the China National Knowledge Infrastructure (CNKI), VIP Database, Wanfang Database, China Biomedical Literature Database (CBM), PubMed, Cochrane Library, Embase, Medline, and Web of Science, to retrieve literature on risk factors for cognitive impairment in patients with coronary heart disease (CHD). Searches were run from database inception to July 2025. After literature screening, data extraction and quality assessment, a meta-analysis was performed using Stata 16.0 and RevMan 5.4 software.

**Results:**

A total of 22 studies were included, involving 9,207 patients. The prevalence of cognitive impairment in CHD patients was 36.6% [95% CI (27.6%–45.7%)]. Meta-analysis and systematic review revealed that age [OR: 1.10 (1.04–1.17), *P* = 0.001], history of stroke [OR: 1.99 (1.09–3.62), *P* = 0.02], smoking [OR: 3.05 (2.00–4.66), *P* < 0.001], diabetes [OR: 2.31 (1.22–4.36), *P* = 0.01], depression [OR: 1.67 (1.24–2.26), *P* < 0.001], and anxiety [OR: 2.03 (1.54–2.69), *P* < 0.001] were significant risk factors for cognitive impairment in CHD patients. In contrast, no significant correlations were observed between cognitive impairment and education level, hypertension, systolic blood pressure, or HDL-C.

**Conclusion:**

Cognitive impairment is notably prevalent among patients with coronary heart disease (CHD). Specifically, elderly patients, smokers, those with a history of stroke or diabetes, and individuals with negative emotions such as anxiety and depression face a particularly high risk of developing cognitive impairment. Healthcare professionals should identify these high-risk groups as early as possible, conduct dynamic assessments of changes in their cognitive function, and implement targeted interventions to effectively prevent the onset of cognitive impairment.

**Systematic Review Registration:**

https://www.crd.york.ac.uk/prospero/, identifier (CRD420251106812).

## 1 Introduction

Coronary Heart Disease (CHD) is a chronic cardiovascular disease characterized by the formation of atherosclerotic plaques in the coronary artery intima, leading to stenosis or occlusion of the vascular lumen (1). The prevalence of CHD is increasing annually, posing a significant threat to human health (2). Reports from the American Heart Association show that the prevalence of coronary heart disease in the United States was about 14.5% in 2021 (3), and is expected to reach 18.0% by 2030 (4). Cognitive impairment is a syndrome characterized by acquired and persistent cognitive dysfunction, which leads to a decline in daily living and working abilities and changes in behavior. Existing studies have clearly demonstrated a significant correlation between CHD and the development of cognitive impairment (5). Compared with the general population, the prevalence of cognitive impairment in CHD patients is significantly higher, with a ratio as high as 2.28 times. Cognitive decline weakens the self-management ability of CHD patients, significantly increasing the risk of death. This not only affects the patients' quality of life but also places a heavy burden on individual health, family care, and social medical resources (6, 7). However, despite the association between cognitive impairment and the poor prognosis of CHD, the impact of cognitive impairment on CHD is difficult to accurately assess due to differences in prevalence estimates. This discrepancy may be attributed to several reasons. First, there is no unified standard for the assessment of cognitive impairment. Currently, neuropsychological scales are widely used to evaluate cognitive function in patients, such as the Mini-Mental State Examination (MMSE) and the Montreal Cognitive Assessment (MoCA). Studies have shown (8) that MMSE exhibits high sensitivity in screening for moderate to severe dementia but performs poorly in detecting mild cognitive impairment and early dementia. In contrast, MoCA, due to its longer administration time and susceptibility to the educational level of the test-taker, has certain limitations in practical application. Second, the inaccuracy of prevalence estimates may stem from differences in the quality and quantity of analyzed studies, as well as varying sampling strategies. Third, cognitive changes are dynamically evolving, and different measurement time points often lead to differences in prevalence estimates. Research indicates that cognitive impairment is highly prevalent in CHD patients one week after surgery, but cognitive function may improve to some extent during the three-month recovery period post-surgery (9).

In addition to clarifying the prevalence of cognitive impairment in CHD patients, accurately identifying factors associated with cognitive function changes is crucial for maintaining cognitive health, early detection, and prevention of cognitive impairment (10). The occurrence of cognitive impairment is influenced by multiple factors. Wang et al. (10) identified age as an important predictor of cognitive impairment in CHD patients, with risk increasing with age. Furthermore, Pan et al. (11) confirmed that in addition to age, hypertension, hyperglycemia, and high Gemini scores are also independent risk factors for cognitive dysfunction in CHD patients. Although these studies have explored the risk factors for cognitive decline, their results are often based on small sample sizes, leading to conflicting outcomes. For instance, Zhou et al. (12) found a close association between smoking and cognitive dysfunction in middle-aged and elderly CHD patients. However, Yang et al. (13) did not support the conclusion that smoking history is an independent risk factor for cognitive impairment in CHD patients based on statistical analysis. Therefore, conducting a Meta-analysis of potential risk factors for cognitive impairment in CHD patients can effectively control research bias and enhance the strength of conclusions, providing high-quality evidence-based theoretical support for targeted cognitive impairment prevention measures.

To date, few systematic reviews have provided summary estimates of cognitive impairment in CHD. Existing studies (14, 15) have only confirmed the correlation between CHD and cognitive impairment, but the specific influencing factors between the two remain unclear and require further investigation. This systematic review aims to clarify the summary prevalence of cognitive impairment in patients with CHD, identify related risk factors, and provide evidence-based recommendations for the prevention and treatment of cognitive deficits. These findings are expected to enhance clinical awareness, management efficiency, and treatment quality of such cognitive impairments, ultimately improving the care outcomes and prognosis of patients with CHD and cognitive impairment.

## 2 Methods

### 2.1 Search strategy

We conducted a comprehensive search of PubMed, Cochrane Library, Web of Science, Embase, Medline, China National Knowledge Infrastructure (CNKI), Wan fang Data, VIP Database, and Chinese Biomedical Literature Database (CBM) from their inception to July 2025. Our search protocol has been registered on the PROSPERO platform with the registration number: CRD420251106812. Additionally, we performed a backward citation search of the references of the included studies. The search terms included “coronary atherosclerotic heart disease”, “myocardial infarction”, “angina pectoris”, “CHD”, “cognitive impairment”, “cognitive decline”, “risk factors”, and “influencing factors”. All retrieved studies were strictly screened to ensure the comprehensive collection of eligible research.

### 2.2 Criteria

Inclusion criteria: (1) The study subjects were adults (≥18 years old) with a confirmed diagnosis of CHD; (2) Integrate the Prevalence rate and/or influencing factors of cognitive impairment; (3) The study design was a cohort study, case-control study, or cross-sectional study.

Exclusion criteria: (1) The original data could not be obtained or transformed; (2) Duplicate publications or articles whose full text could not be obtained; (3) non-Chinese or non-English articles.

### 2.3 Study selection and data extraction

Two researchers independently performed literature screening and data extraction in accordance with predefined inclusion and exclusion criteria. Any disagreements were resolved through discussion or by consulting a third researcher. The extracted information included the first author's name, publication year, country, study design, sample size, mean age of patients, other factors adjusted in the model, measurement methods, and outcomes.

### 2.4 Quality assessment

Two researchers independently assessed the quality of the included studies and cross-validated the results. For cross-sectional studies, the Agency for Healthcare Research and Quality (AHRQ) bias risk scale (16) was adopted. Studies with scores of 0–3 were rated as low quality, 4–7 as moderate quality, and 8–11 as high quality. For cohort and case-control studies, the Newcastle-Ottawa Scale (NOS) (17) was used for quality assessment. A total score of 9 was defined as high quality.

### 2.5 Statistical analysis

The extracted data were analyzed using Stata 16.0 and RevMan 5.4 software. Heterogeneity among studies was assessed using Cochrane's Q statistic, and the degree of heterogeneity was evaluated using the I² statistic: if *P* > 0.10 and *I*^2^ ≤ 50%, the heterogeneity was considered acceptable, and a fixed-effects model was used for analysis; if *P* ≤ 0.10 and *I*^2^ > 50%, significant heterogeneity was indicated, and a random-effects model was applied. The proportion of participants diagnosed with cognitive impairment was extracted from all included studies to calculate the pooled prevalence. To evaluate the risk factors for cognitive impairment in CHD patients, odds ratios and their corresponding 95% confidence intervals were extracted, and all eligible available data from the literature were summarized blication bias was assessed using a funnel plot for visual analysis and verified for symmetry using Egger's test.

## 3 Results

### 3.1 Inclusion of studies

**Figure 1:**
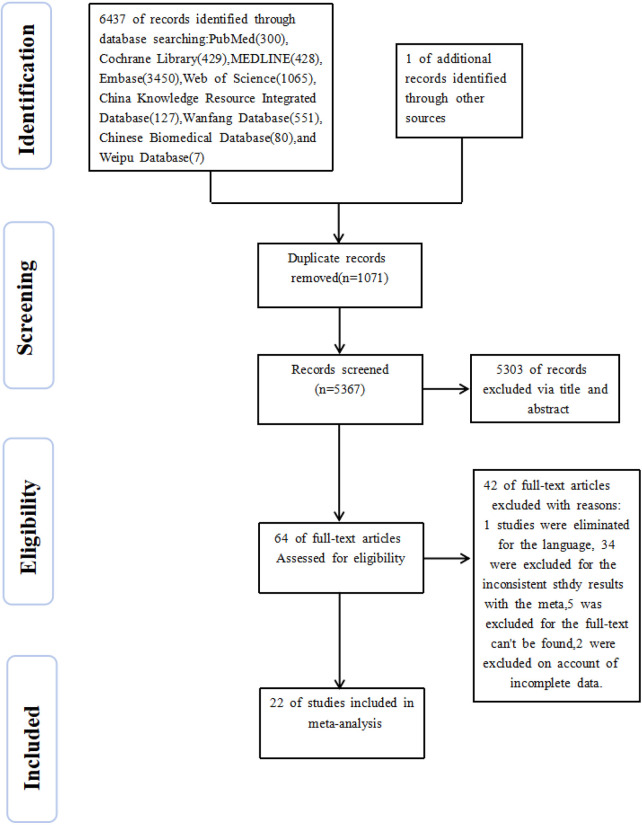
Flowchart of literature screening.

A total of 6,437 articles were identified through the search, and 1 additional article was obtained from other sources. Of these, 1,071 were duplicates and were removed. After preliminary screening of titles and abstracts, 64 articles remained. After reading the full texts, 34 articles were excluded for being irrelevant to the study content, 2 for incomplete data, 5 for unavailability, and 1 for being non-Chinese or non-English. Ultimately, 22 articles met the inclusion criteria and were included in this meta-analysis, comprising 14 Chinese studies and 8 English studies (Figure 1). The combined kappa statistic for inter-rater reliability was 0.88, indicating excellent consistency between the two independent reviewers during study selection.

### 3.2 Study characteristic

Table 1 summarizes the basic features of the 22 included studies. These studies were published between 2015 and 2025, with 19 conducted in China, one in the United States, one in the United Kingdom, and one in Serbia. The methodological evaluation system confirmed that nine studies (10, 18, 19, 21, 22, 25, 31, 34, 35) were high-quality papers and thirteen studies (11–13, 20, 23, 24, 26–30, 32, 33) were medium-quality papers. five studies (18, 19, 21, 31, 35) were prospective cohort studies, and only six studies (10, 18, 24, 25, 28, 34) had a sample size exceeding 300 participants. Additionally, one study (18) was a multicenter observational cohort study, and none of the studies reported the 95% confidence interval for the prevalence of cognitive impairment.

**Table 1:** Characteristics of all included studies.

First author (year)	Country	Study types	Sample size	Sex (M: F)	Sample age	Evaluation tool	Prevalence	Event	*n*	Influence factor	OR	95% CI	Quality assessment
Zheng et al. (18)	China	cohort study	3,244	1,899: 1,345	70.41 ± 4.37	MMSE MoCA	63.04%	2,045	3,244	age	1.03	1.01–1.04	7
										HbA1c ≥ 6.5%	1.79	1.41–2.26	
										education level	0.23	0.15–0.33	
										history of stroke	1.35	1.00–1.82	
Gu et al. (19)	UK	cohort study	271	169: 102	80.5 ± 4.8	MoCA	35%	74	211	recurrent MI	3.19	1.18–8.63	7
										smoking	2.17	1.20–3.92	
										low physical activity	2.08	1.12–3.86	
										slow walking	2.4	1.02–5.67	
Mao (20)	China	Cross- sectional study	249	82: 167	40–75	MMSE	32.93%	82	249	age	0.377	0.248–0.572	7
										hypertension	10.276	1.315–80.317	
										depression	3.971	2.269–6.950	
										HDL-C	0.036	0.001–0.954	
Fu et al. (21)	China	cohort study	268	147: 121	CI 60.27 ± 12.41 NCI 53.73 ± 13.18	MoCA	30.60%	82	268	Age > 65	1.154	1.021–1.287	7
										hypertension	1.097	1.015–1.179	
										LVEF < 50%	1.325	1.173–1.477	
										serum 25(OH)D3 < 31.41 ng/mL	1.229	1.076–1.382	
										surgical time >60 min	1.248	1.084–1.412	
Liu et al. (22)	China	Case-control study	223	175: 48	CHD 73.04 ± 10.22 NCHD 64.11 ± 9.27	MMSE	40.36%	90	223	age	1.086	1.047–1.127	7
										fatigue	2.537	1.068–6.026	
										sleep disorders	1.76	1.106–2.799	
Shang et al. (23)	China	Cross-sectional study	208	117: 91	65.68 ± 8.76	MMSE	16.30%	34	208	LVEF	0.928	0.882–0.976	7
Bai et al. (24)	China	Cross-sectional study	448	323: 125	CI 62.86 ± 5.21 NCI 58.77 ± 5.63	MoCA	41.29%	185	448	age	1.128	1.081–1.177	4
										fasting blood glucose	2.216	1.717–2.861	
										systolic blood pressure	1.038	1.023–1.053	
										number of vascular lesions	1.166	1.086–1.567	
Pan et al. (11)	China	Cross-sectional study	163	97: 66	CI 51.84 ± 9.46 NCI 64.69 ± 8.58	MMSE	35.60%	58	163	age	2.728	0.459–1.154	7
										41 ≤ Gensini<64	5.461	1.890–15.775	
										Gensini≥64	40.640	7.991–206.689	
										hypertension	2.445	0.621–3.366	
										diabetes	1.252	0.538–2.914	
Ma et al. (25)	China	Case-control study	445	252: 193	CI 64.54 ± 9.43 NCI 59.54 ± 8.43	MMSE	12.13%	54	445	age	1.073	1.026–1.121	8
										hypertension	4.885	2.193–10.881	
										ICU stay duration	1.025	1.009–1.041	
										high SIRS score	3.083	1.996–4.761	
										high serum TNF-α level	1.173	1.099–1.252	
										Severe degree of coronary artery stenosis	5.107	1.729–15.081	
Zhang (26)	China	Cross-sectional study	130	88: 42	——	MoCA	26.90%	35	130	age≥65	2.123	1.150–3.921	6
										postoperative hypoxemia	3.023	1.128–8.102	
										preoperative use of dexmedetomidine	0.698	0.540–0.902	
Chen (27)	China	Cross-sectional study	160	120: 40	NCI 58.09 ± 13.17 MCI 59.45 ± 8.55 MSCI 72.36 ± 8.59	TICS-m	47.50%	76	160	age	2.162	1.362–16.495	5
										years of education	0.314	0.129–0.769	
										time from onset to vessel recanalization	2.604	1.086–6.241	
										myoglobin	2.421	1.170–5.009	
										cumulative IABP time < 72h	0.459	0.250–0.841	
										cumulative IABP time ≥ 72 h	0.518	0.349–0.768	
Wu et al. (28)	China	Cross-sectional study	420	267: 153	60.28 ± 12.53	MoCA	28.10%	118	420	age	1.792	1.114–5.027	6
										diabetes	2.129	1.332–6.223	
										atrial fibrillation	1.899	1.223–5.121	
										systolic blood pressure	1.771	1.129–4.278	
										HDL-C	0.760	0.419–1.086	
										depression	1.491	1.346–5.221	
Ma et al. (29)	China	Cross-sectional study	110	62: 48	65.23 ± 4.98	MMSE	23.64%	26	110	age > 70	2.214	1.233–3.879	5
										anxiety	1.967	1.129–2.998	
										Rate of change of rSO2 > 30%	2.112	1.182–3.657	
										cardiopulmonary bypass	2.010	1.253–3.509	
										ICU stay duration > 7 h	2.224	1.339–4.231	
Zhe (30)	China	Cross-sectional study	196	102: 94	65–75	MoCA	67.90%	133	196	years of education	0.86	0.77–0.91	7
Su et al. (31)	China	Case-control study	141	83: 58	60–81	MoCA	22.80%	26	114	subjective memory impairment	11.18	2.63–47.5	8
Chen et al. (32)	China	Cross-sectional study	80	53: 27	≥60	MoCA	67.50%	54	80	age	1.458	1.220–1.742	6
										male	0.116	0.035–0.383	
										anxiety	2.056	1.487–2.843	
										depression	1.300	1.113–1.519	
										B-type natriuretic peptide	1.002	1.000–1.003	
										6 MWD	0.980	0.968–0.993	
										VO_2max_	0.629	0.452–0.874	
										Taking ACEI or ARB	0.072	0.015–0.335	
zhao (33)	China	Cross-sectional study	120	71: 49	45–70	MoCA	44.20%	53	120	smoking	3.154	1.30–7.63	5
										age:56–65	4.375	1.55–12.32	
										age:66–75	4.276	1.27–14.35	
										Gensini ≥ 50	5.11	1.51–17.35	
Saczynski et al. (34)	US	observational study	1,521	1,019: 502	62 ± 11	TICS	20%	304	1,521	history of stroke	2.5	1.0–5.8	8
Zhou et al. (12)	China	Cross-sectional study	268	178: 80	66.62 ± 9.75	MMSE	23.50%	63	268	smoking	5.728	2.518–13.028	6
										age	1.085	1.046–1.125	
										diabetes	4.668	2.075–10.502	
										history of stroke	3.248	1.425–7.408	
Dikić et al. (35)	Serbia	cohort study	82	60: 22	CI 63.12 ± 6.56 NCI 57.34 ± 7.39	MMSE	20.70%	17	82				9
Wang et al. (10)	China	Cross-sectional study	400	248: 152	70.86 ± 8.74	MoCA	55%	220	400				8
Yang et al. (13)	China	Cross-sectional study	60	45: 15	49.7 ± 19.7	TICS-m	55%	33	60				7

MMSE, mini-mental state examination; MoCA, montreal cognitive assessment; TICS, telephone interview of cognitive status; CI, cognitive impairment; NCI, non-cognitive impairment.

### 3.3 Meta-analysis of results

#### 3.3.1 Prevalence of cognitive impairment

**Figure 2:**
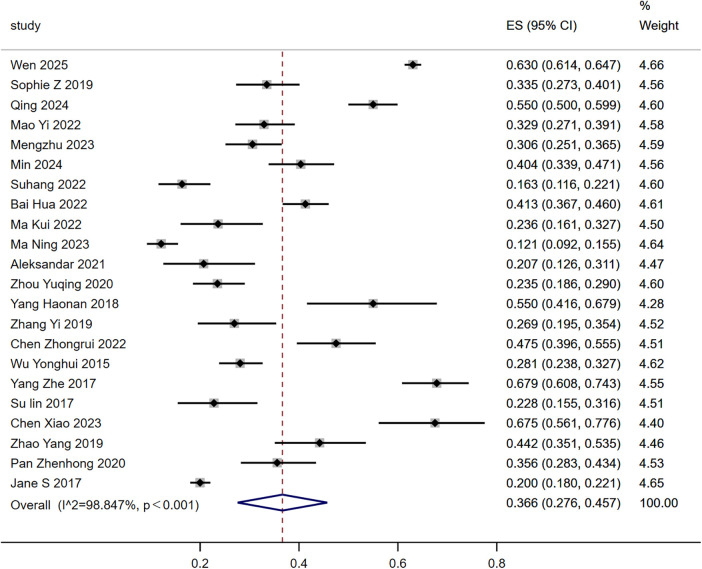
Forest plot of the prevalence of cognitive impairment in patients with coronary heart disease.

Among the 22 studies included in the meta-analysis, the prevalence of cognitive impairment in CHD patients ranged from 12.13% to 67.9%. Meta-analysis of all data revealed significant heterogeneity among studies (*P* < 0.001, *I*^2^ = 98.85%). The random-effects model analysis showed that the incidence of cognitive impairment in CHD patients was 36.6% [95% CI (0.276–0.457)], as shown in Figure 2.

#### 3.3.2 Subgroup analysis

A subgroup analysis of the prevalence rate was performed according to research design, measurement tools, and clinical context. The results of this meta-subgroup analysis are shown in Table 2.

**Table 2:** Subgroup analysis of cognitive impairment prevalence in CHD patients.

Subgroup	Study	heterogeneity test		effects model	Prevalence rate (%)	95% CI	Meta regression	
		*I*^2^ (%)	P				95% CI	*P*
Measuring tool							−0.14 (−0.44,0.16)	0.34
MoCA	10	95.7	<0.001	random	41.6	0.32–0.51		
MMSE	8	93.2	<0.001	random	25.5	0.18–0.33		
TICS	3	97.2	<0.001	random	40.3	0.17–0.64		
Study design							−0.10 (−0.46,0.26)	0.57
Cross-sectional								
Study	14	95.9	<0.001	random	40.1	0.32–0.48		
Cohort study	5	99.6	<0.001	random	33.6	0.10–0.57		
Case-control study	3	96.8	<0.001	random	25.0	0.07–0.43		
Clinical context							−0.09 (−0.68,0.51)	0.75
ACS	5	92.0	<0.001	random	30.1	0.22–0.38		
Post-CABG/PCI	6	95.3	<0.001	random	28.8	0.18–0.40		

MMSE, mini-mental state examination; MoCA, montreal cognitive assessment; TICS, telephone interview of cognitive status; ACS, acute coronary syndrome; post-CABG = post–coronary artery bypass grafting; post-PCI, post–percutaneous coronary intervention.

#### 3.3.3 Meta-regression

Using the prevalence of cognitive impairment in patients with coronary heart disease as the dependent variable, and study design, clinical setting, and cognitive impairment assessment tools as independent variables, meta-regression was performed. The results indicated that none of these variables were significant moderators of the prevalence of cognitive impairment.

#### 3.3.4 Sensitivity analysis

**Figure 3:**
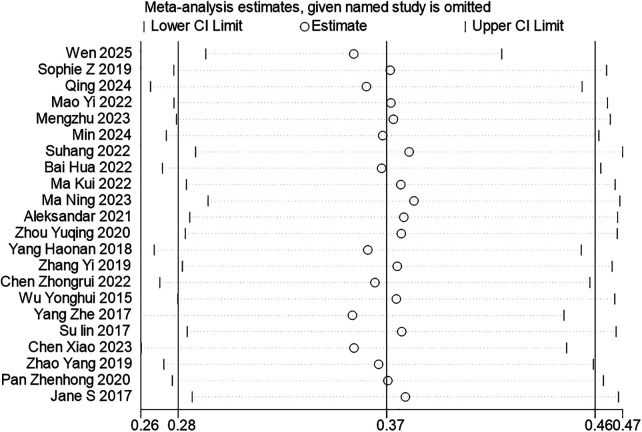
Sensitivity analysis plot for pooled prevalence.

In this study, a stepwise exclusion approach was used for the sensitivity analysis (Figure 3). The results showed that after omitting each included study individually, the pooled effect size for the prevalence of cognitive impairment remained within the original confidence interval, suggesting that the findings were robust.

#### 3.3.5 Factors associated with cognitive impairment

**Figure 4:**
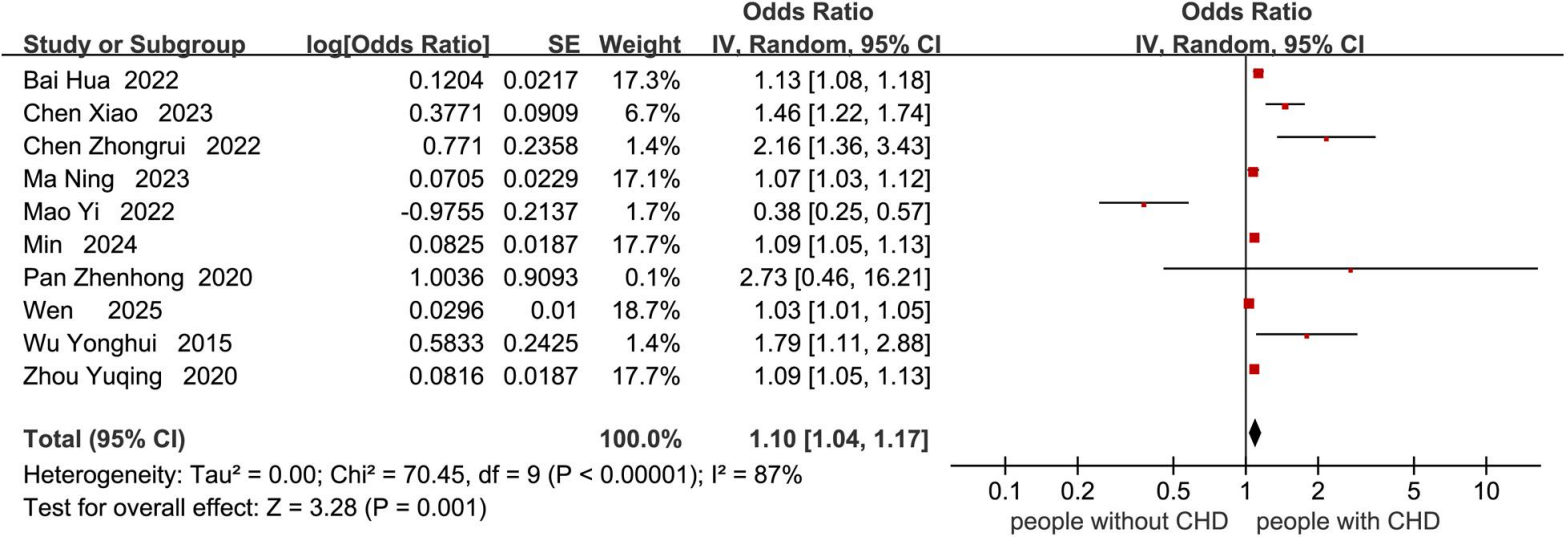
Forest plot of age.

**Figure 5:**
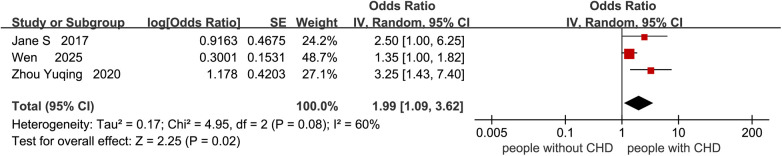
Forest plot of history of stroke.

**Figure 6:**
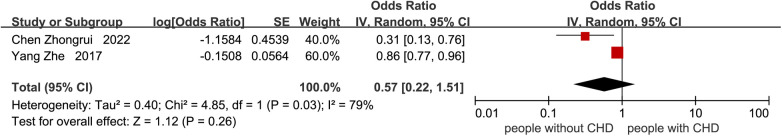
Forest plot of years of education.

**Figure 7:**
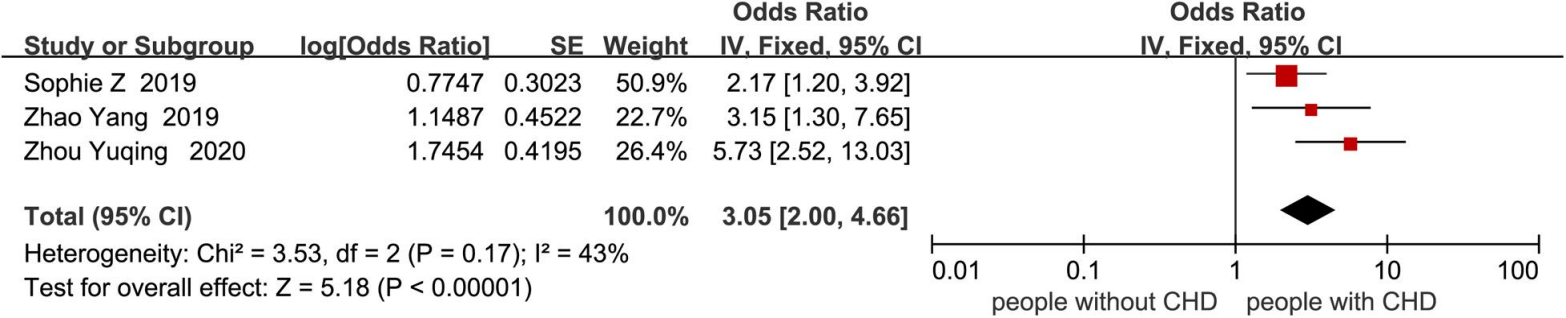
Forest plot of smoking.

**Figure 8:**
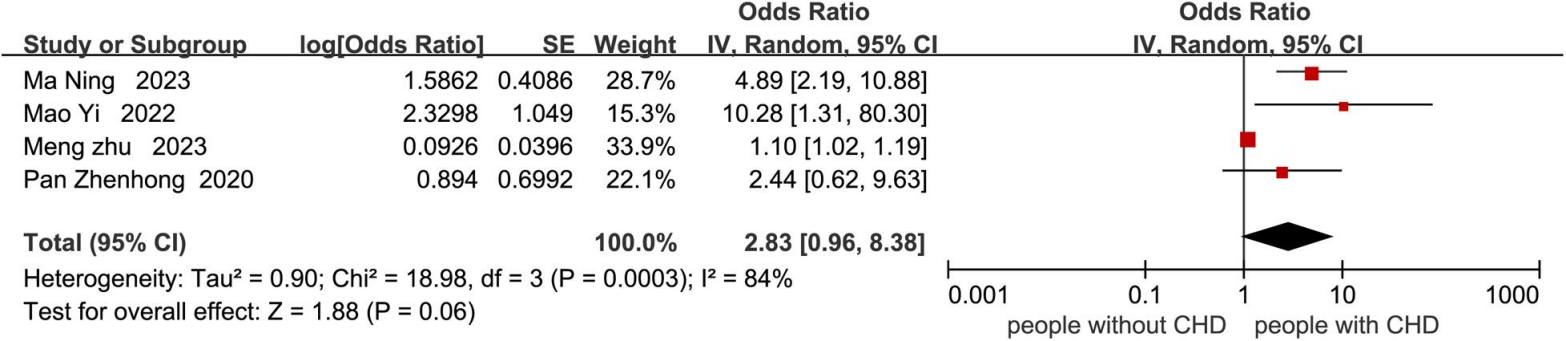
Forest plot of hypertension.

**Figure 9:**
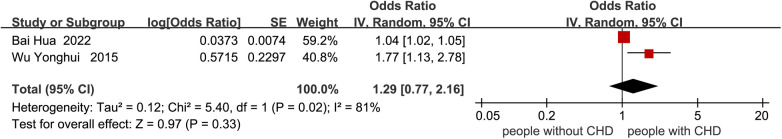
Forest plot of systolic blood pressure.

**Figure 10:**
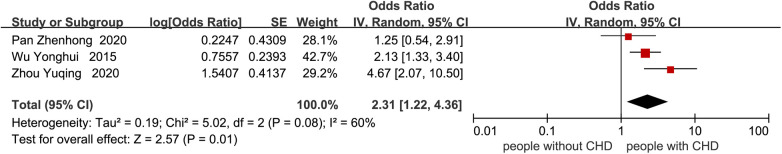
Forest plot of diabetes.

**Figure 11:**
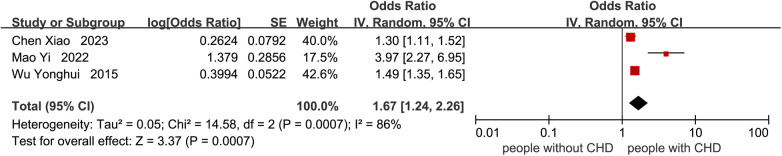
Forest plot of depression.

**Figure 12:**
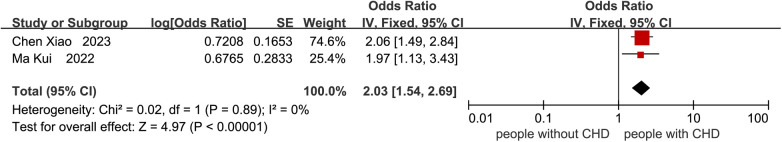
Forest plot of anxiety.

**Figure 13:**
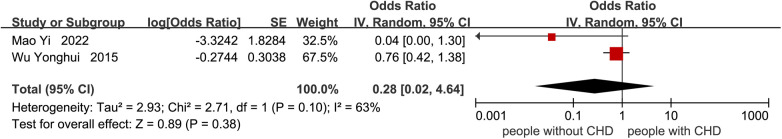
Forest plot of HDL-C.

Nineteen studies reported factors influencing cognitive impairment. Four studies (21, 26, 29, 33) related to age were not included in the meta-analysis of age due to subgroup analysis with different age ranges. Three studies (11, 25, 33) related to the degree of coronary stenosis were not included in the meta-analysis due to different scoring ranges in each study. One study (18) related to education level was not included in the meta-analysis of years of education due to the use of educational level rather than years of education. Two studies (21, 23) related to LVEF were not included in the meta-analysis due to different subgroup analyses. Factors such as male gender, atrial fibrillation, B-type natriuretic peptide, subjective memory impairment, number of vascular lesions, fasting blood glucose, fatigue, sleep disorders, serum 25(OH)D3 < 31.41 ng/mL, recurrent MI, HbA1c ≥ 6.5%, low physical activity, slow walking, surgical time >60 min, ICU stay duration, high SIRS score, high serum TNF-α level, postoperative hypoxemia, preoperative use of dexmedetomidine, time from onset to vessel recanalization, myoglobin, cumulative IABP time, cardiopulmonary bypass, 6MWD, VO2max, taking ACEI or ARB were not included in the meta-analysis due to the fact that fewer than two studies were conducted. Therefore, the meta-analysis compared cognitive decline in CHD patients based on age, history of stroke, years of education, smoking, hypertension, systolic blood pressure, diabetes, depression, anxiety, and HDL-C (Figures 4–13). After adjusting for mixed factors, age [OR: 1.10 (1.04–1.17), *P* = 0.001], history of stroke [OR: 1.99 (1.09–3.62), *P* = 0.02], smoking [OR: 3.05 (2.00–4.66), *P* < 0.001], diabetes [OR: 2.31 (1.22–4.36), *P* = 0.01], depression [OR: 1.67 (1.24–2.26), *P* < 0.001], and anxiety [OR: 2.03 (1.54–2.69), *P* < 0.001] were significantly associated with an increased risk of cognitive impairment in CHD patients. In contrast, education level (*P* = 0.26), hypertension (*P* = 0.06), systolic blood pressure (*P* = 0.33), and HDL-C (*P* = 0.38) were not statistically significant.

### 3.4 Publication bias

**Figure 14:**
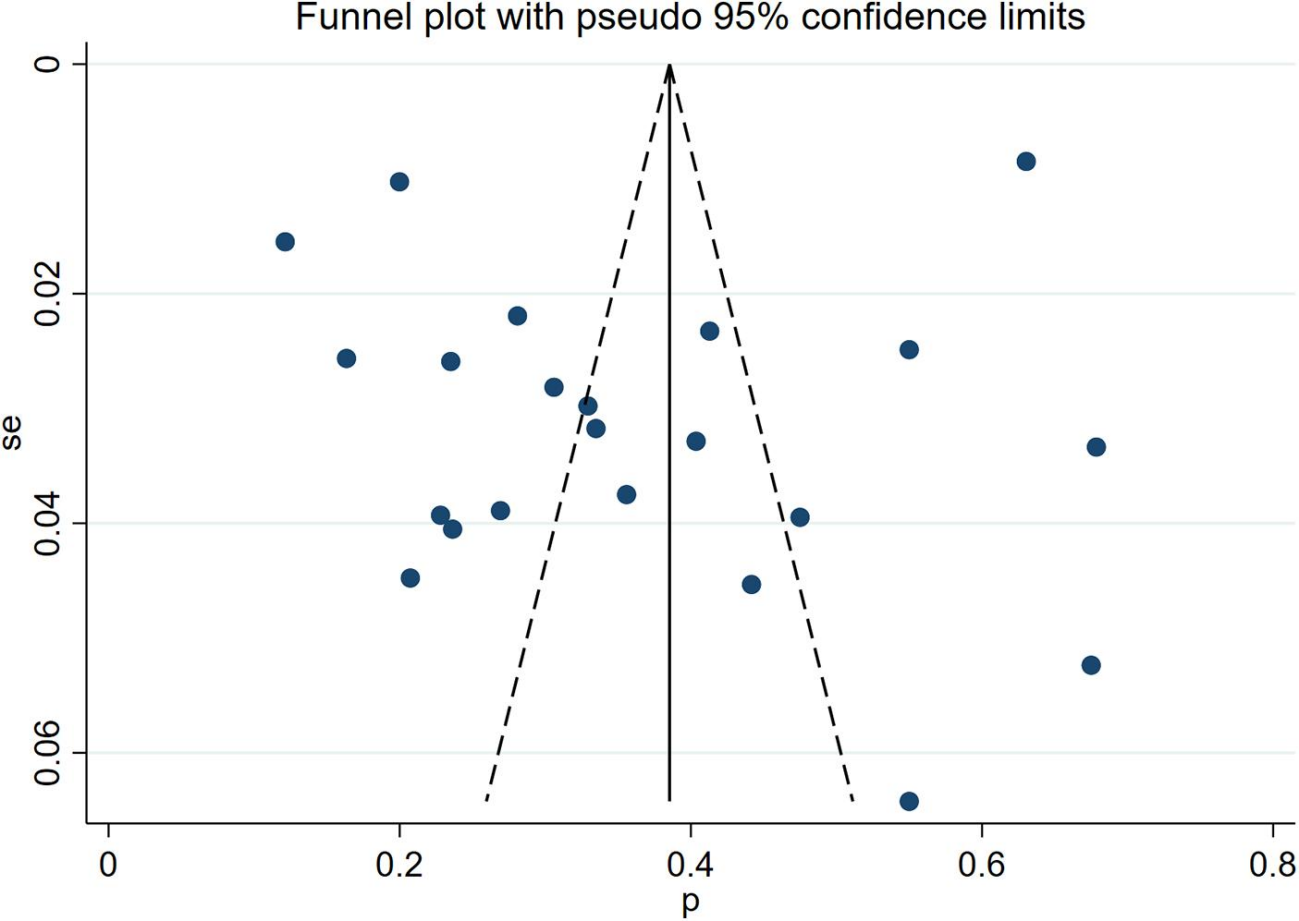
Funnel plot.

The symmetry of the funnel plot (Figure 14) indicates no publication bias among the studies, which was further confirmed by Egger's weighted regression test (*P* = 0.440).

## 4 Discussion

This study conducted a comprehensive systematic review and meta-analysis of 22 studies, involving 9,207 patients diagnosed with CHD, with a prevalence of cognitive impairment reaching 37%, significantly higher than that observed in patients with asthma (36). This elevated prevalence is closely related to the unique pathophysiological mechanisms of CHD. On one hand, the decreased cardiac output in CHD patients can directly lead to cerebral hypoperfusion, reducing the oxygen and energy supply to brain cells, thereby causing brain cell damage and ultimately resulting in cognitive dysfunction (37). On the other hand, pathological factors such as myocardial ischemia and reperfusion injury caused by CHD can continuously exacerbate the body's oxidative stress levels. Under high oxidative stress conditions, the interaction between excessive reactive oxygen species and mitochondrial dysfunction forms a synergistic effect, triggering abnormal expression of pro-inflammatory mediators, vascular endothelial damage, and vascular remodeling. These pathological changes ultimately manifest as lipid peroxidation, protein oxidation, and nucleic acid damage, directly impacting the structure and function of neurons and leading to cognitive decline (38). Moreover, the results of this study indicate a wide fluctuation in the incidence of cognitive impairment among the surveyed population, ranging from a minimum of 12.13% (25) to a maximum of 67.9% (30). Notably, both the highest and lowest incidences were found in China. The potential determinants for the significant prevalence differences within the same country are as follows:1. Significant Sample Size Differences: Among the included Chinese studies, the sample size ranged from 80 to 3,244 participants, contributing to the observed heterogeneity in results. 2. Regional Differences: The majority of the included studies were single-center, with significant variations in cognitive impairment prevalence across different regions. For instance, the prevalence was as high as 67.9% in Harbin, while it was only 12.13% in Zhengzhou. This may be related to differences in the selection of study subjects. 3. Inconsistent Gender Ratios of Participants: In the included studies, the proportion of male participants was disproportionately high. Research has shown (32) that there are differences in cognitive levels between males and females. Compared to females, male patients often engage in more social labor, have more frequent external contacts, and receive richer effective information stimulation, thus obtaining more opportunities for cognitive function exercise and performing better in cognitive ability assessments. This gender imbalance may lead to an underestimation of the overall prevalence of cognitive impairment in the studies. The subgroup analyses in this study revealed the following: (1) Regarding assessment tools, the prevalence of cognitive impairment in patients with coronary heart disease detected by the MMSE was significantly lower than that detected by the MoCA and TICS. This may be attributed to differences in the assessment dimensions of these scales. The MMSE performs well in identifying moderate to severe cognitive impairment but has relatively low sensitivity for screening mild cognitive impairment. (2) In terms of clinical characteristics, the prevalence of cognitive impairment in patients with coronary heart disease following coronary artery bypass grafting (CABG) or percutaneous coronary intervention (PCI) was similar to that in patients with acute coronary syndrome (ACS), which is consistent with the findings reported by previous studies (39).

The results of this study indicate that age is a risk factor for cognitive impairment in CHD patients, consistent with the findings of Zhao et al. (40). Previous studies have confirmed (33) that CHD patients aged 56–75 years have a risk of cognitive impairment more than three times higher than those aged 45–55 years. This may be related to the decline in central cholinergic system function in older patients. With increasing age, the cholinergic system function in central areas related to cognitive function gradually declines, with a reduction in cholinergic neurons and the occurrence of neuronal atrophy and degenerative changes, leading to a decline in core cognitive functions such as executive ability and memory (26). Moreover, compared to middle-aged and young patients, elderly CHD patients are more prone to characteristic intracranial pathological changes driven by the aging process, such as brain structural remodeling, a reduction in brain neurons, and downregulation of neurotransmitter levels. These changes can directly interfere with the normal information reception, integration, and processing pathways of the brain, significantly weakening the patients' cognitive function reserve and inducing cognitive impairment (41). Although this meta-analysis integrated ten studies, none of the included studies provided a clear definition for “age”. Therefore, future studies should strictly follow the World Health Organization's age classification standards for stratified analysis to ensure reliable evidence for the development of precise clinical interventions. In addition, while nursing staff should prioritize monitoring cognitive function in elderly patients with coronary artery disease (CAD), greater attention must also be paid to younger and middle-aged populations. Evidence indicates that the prevalence of cognitive impairment reaches 39.19% among young and middle-aged patients with acute myocardial infarction (42). Therefore, it is recommended that cognitive function assessment be integrated into the routine nursing evaluation of all patients with CAD. For individuals at high risk of cognitive decline, intensive longitudinal monitoring and structured cognitive training protocols should be implemented. These targeted nursing interventions help modulate brain structure and function, thereby slowing the progression of cognitive impairment.

The results of this study indicate that smoking is a risk factor for cognitive impairment in patients with CHD, consistent with a 5.5-year prospective study from the Netherlands (43). Previous research has found (44) that long-term smoking in CHD patients can lead to the generation of reactive oxygen species and oxidative damage, resulting in endothelial nitric oxide synthase dysfunction. This further exacerbates cerebral vascular and blood-brain barrier damage, induces inflammation in the brain, and causes neuronal degeneration, ultimately leading to cognitive dysfunction. Additionally, toxic particles from smoke can target and impair mitochondrial function in CHD patients. The resulting energy depletion in neurons can lead to neuronal death and subsequently affect cognitive function (45). However, a study by Ren and Deng (46) suggested that mild and moderate smoking might be protective factors against cognitive impairment, which may be related to nicotine intake. Research has shown (34) that the cognitive effects of nicotine are dose-dependent. Low doses of nicotine can improve cognitive function, while higher doses may have no significant beneficial effects or even cause harm. The studies included in this analysis only reported categorical smoking status, which precluded a quantitative evaluation of the dose–response relationship between smoking exposure and cognitive impairment. However, Previous studies (47) adopted the “pack-year” metric to quantify cumulative smoking exposure and examine its association with cognitive decline. Their results indicated that, compared with never smokers, patients with <19 pack-years had a mean reduction of 1.05 points in the total MMSE score; those with 19–47 pack-years showed a 1.16-point decline, whereas individuals with >47 pack-years experienced a 1.17-point decline. These findings suggest that higher cumulative smoking exposure exacerbates cognitive deterioration. Future studies could use “pack-years” as an indicator to quantify cumulative smoking exposure and conduct stratified analyses to further clarify the specific impact of smoking behavior on cognitive function among different smoking subgroups of patients with CHD.

The results of this study also show that comorbidities are a risk factor for cognitive impairment in CHD patients, in line with the findings of Yang et al. (48). CHD patients often have multiple comorbidities, such as stroke and diabetes. When multiple diseases coexist, they interact and have a synergistic effect on vascular endothelial cell damage, exacerbating cerebral ischemia and hypoxia and creating a vicious cycle that further impairs cognitive function (49). Moreover, the coexistence of multiple diseases can further intensify the inflammatory response in CHD patients, leading to elevated levels of inflammatory markers such as C-reactive protein (CRP), tumor necrosis factor-α (TNF-α), and interleukins. These inflammatory factors can cross the blood-brain barrier and enter the central nervous system, activating neuroinflammatory pathways and promoting the deposition of β-amyloid and tau protein phosphorylation, thereby affecting cognitive function (50). Additionally, patients with multiple comorbidities often require polypharmacy to manage their underlying conditions. Research has indicated (51) that the more medications a patient takes, the greater the medication burden and the higher the risk of drug interactions. While a single drug may not cause significant side effects or only produce mild reactions, polypharmacy can lead to additive side effects and pharmacodynamic interactions (e.g., anticholinergic drugs exacerbating central nervous system depression), potentially inducing drug-related cognitive impairment. In clinical practice, healthcare providers should enhance comprehensive management guidance for CHD patients with comorbidities and strengthen the standardized control of polypharmacy to reduce the risk of cognitive impairment.

The results of this study also indicate that anxiety and depression are risk factors for cognitive impairment in CHD patients, consistent with the findings of Lin et al. (52). Previous studies have shown (53, 54) that chronic anxiety and depression can continuously activate the “hypothalamic-pituitary-adrenal axis”, leading to excessive cortisol secretion. Long-term high levels of cortisol can induce significant changes in hippocampal cytoskeletal proteins, resulting in impaired structural integrity and functional abnormalities of hippocampal neurons, ultimately leading to cognitive decline. Moreover, anxiety and depression can severely erode sleep quality, causing sleep disorders such as difficulty falling asleep and insomnia. Poor sleep quality can further lead to neurotransmitter imbalances and cortical dysfunction, disrupting the normal information processing and memory storage mechanisms in the brain and causing significant cognitive impairment (22). Therefore, medical professionals should not only focus on patients’ physical health but also enhance the evaluation of their psychological status. They should encourage patients to express their genuine inner feelings and facilitate appropriate emotional release, thereby alleviating emotional disturbances and lowering the risk of cognitive impairment.

This study found that while factors such as reduced LVEF, postoperative hypoxemia, and sleep disturbances were excluded from the meta-analysis due to the limited number of eligible studies, existing evidence supports their association with cognitive impairment. Reduced LVEF reflects impaired left ventricular systolic function, which leads to decreased cardiac output and cerebral hypoperfusion, ultimately resulting in neuronal metabolic dysfunction and microcirculatory stasis, thereby contributing to cognitive decline (55). Previous studies (26) have demonstrated that coronary heart disease patients with postoperative hypoxemia face a threefold higher risk of cognitive impairment compared with those without hypoxemia, a link that may be closely related to neurotoxicity mediated by cerebral hypoxia. Furthermore, a study (56) noted that sleep deprivation impairs the brain's efficiency in clearing metabolic waste during deep slow-wave sleep, promoting abnormal amyloid-β deposition and neurotoxicity, which in turn accelerates cognitive decline. Future large-scale, multicenter prospective cohort studies are warranted to further validate the causal relationship between these factors and cognitive impairment and to explore potential intervention targets.

In summary, advanced age, smoking, multimorbidity, anxiety, and depression were identified as risk factors for cognitive impairment in patients with coronary heart disease. These factors do not act independently but interact with each other closely. Elderly patients often present with multiple chronic comorbidities, and the substantial disease burden predisposes them to negative psychological conditions such as anxiety and depression (57). Such negative emotions may further contribute to unhealthy behaviors including smoking, which exacerbates vascular endothelial injury and accelerates atherosclerosis. Sustained vascular damage and systemic inflammation subsequently aggravate both coronary heart disease and cognitive decline, ultimately forming a vicious cycle among physical diseases, psychological disorders, and unhealthy lifestyles. Therefore, comprehensive screening and integrated interventions targeting these interrelated factors are warranted in clinical practice.

A key strength of this study is its rigorous methodology, including an exhaustive literature review of nine different databases, covering both English and Chinese academic studies. This extensive investigation enhances the accuracy of data on cognitive impairment in CHD patients and considers various factors influencing cognitive impairment, such as demographic factors, psychological factors, and comorbidities. To our knowledge, this study offers considerable value, as it not only precisely quantifies the prevalence of cognitive impairment in CHD patients but also conducts a detailed assessment of potential influencing factors. However, our study has certain limitations. First, the included studies were limited to English and Chinese, excluding articles published in other languages, and most studies were conducted in China, limiting the comprehensiveness of the included literature. However, the included studies were mostly of high quality, yielding precise and reliable results. Second, some influencing factors were not subjected to meta-analysis due to data scarcity, but our study comprehensively considered factors affecting cognitive impairment at different stages of CHD, covering the entire disease cycle as much as possible. In addition, only studies reporting odds ratios (OR) with 95% confidence intervals were included, which may introduce selection bias by excluding investigations with incompletely reported effect estimates. Nevertheless, this stringent criterion ensured methodological consistency and comparability across studies, providing a reliable foundation for the quantitative pooling of results. Finally, the included literature did not cover unpublished articles and studies, but we searched nine major databases to ensure the original literature was as comprehensive as possible. Furthermore, this study is subject to notable geographic bias. Approximately 86% of the included participants were from China, with only a very limited number of studies conducted in other regions, which may restrict the external validity of our findings. Given the differences in lifestyle, genetic background, and healthcare resources between the Chinese population and cohorts from regions such as Europe and North America, caution should be exercised when generalizing our conclusions to non-Chinese populations, and further validation in diverse cohorts is warranted. Nevertheless, substantial heterogeneity (*I*^2^ > 90%) was detected across all analyses, and meta-regression did not identify any significant moderators contributing to the observed variability. Accordingly, the pooled prevalence estimate should be interpreted with extreme caution. This pooled estimate has limited clinical interpretability and should not be considered a precise summary measure. Given the high level of unexplained heterogeneity, readers are advised to avoid overinterpreting the pooled result. Considering these limitations, more in-depth research is necessary in the future to provide a more comprehensive perspective for strategies to prevent cognitive impairment in CHD patients.

## 5 Conclusion

This study reveals that the prevalence of cognitive impairment in patients with CHD is as high as 36.6%. This elevated prevalence underscores the need to further enhance public and clinical awareness of cognitive impairment in CHD patients. Additionally, the study identifies age, smoking, history of stroke, diabetes, and anxiety/depression as risk factors for cognitive impairment in CHD patients. Based on these findings, researchers and clinicians should intensify early screening efforts, provide comprehensive care for high-risk patients, and implement targeted interventions such as smoking cessation, disease management, and psychological support to lower the risk of cognitive impairment.

## Data Availability

The original contributions presented in the study are included in the article/Supplementary Material, further inquiries can be directed to the corresponding author.
